# Ultrahigh dose rate pencil beam scanning proton dosimetry using ion chambers and a calorimeter in support of first in‐human FLASH clinical trial

**DOI:** 10.1002/mp.15844

**Published:** 2022-07-14

**Authors:** Eunsin Lee, Ana Mónica Lourenço, Joseph Speth, Nigel Lee, Anna Subiel, Francesco Romano, Russell Thomas, Richard A. Amos, Yongbin Zhang, Zhiyan Xiao, Anthony Mascia

**Affiliations:** ^1^ Department of Radiation Oncology University of Cincinnati Cincinnati Ohio USA; ^2^ Cincinnati Children's Hospital Medical Center Cincinnati Ohio USA; ^3^ National Physical Laboratory Medical Science Group Teddington UK; ^4^ Department of Medical Physics and Biomedical Engineering University College London London UK; ^5^ University of Cincinnati Medical Center Cincinnati Ohio USA; ^6^ Istituto Nazionale di Fisica Nucleare Sezione di Catania Catania Italy; ^7^ Faculty of Engineering and Physical Science University of Surrey Guildford Surrey UK

**Keywords:** dosimetry, FLASH, PBS

## Abstract

**Purpose:**

To provide ultrahigh dose rate (UHDR) pencil beam scanning (PBS) proton dosimetry comparison of clinically used plane‐parallel ion chambers, PTW (Physikalisch‐Technische Werkstaetten) Advanced Markus and IBA (Ion Beam Application) PPC05, with a proton graphite calorimeter in a support of first in‐human proton FLASH clinical trial.

**Methods:**

Absolute dose measurement intercomparison of the plane‐parallel plate ion chambers and the proton graphite calorimeter was performed at 5‐cm water‐equivalent depth using rectangular 250‐MeV single‐layer treatment plans designed for the first in‐human FLASH clinical trial. The dose rate for each field was designed to remain above 60 Gy/s. The ion recombination effects of the plane‐parallel plate ion chambers at various bias voltages were also investigated in the range of dose rates between 5 and 60 Gy/s. Two independent model‐based extrapolation methods were used to calculate the ion recombination correction factors *k_s_* to compare with the two‐voltage technique from most widely used clinical protocols.

**Results:**

The mean measured dose to water with the proton graphite calorimeter across all the predefined fields is 7.702 ± 0.037 Gy. The average ratio over the predefined fields of the PTW Advanced Markus chamber dose to the calorimeter reference dose is 1.002 ± 0.007, whereas the IBA PPC05 chamber shows ∼3% higher reading of 1.033 ± 0.007. The relative differences in the *k_s_* values determined from between the linear and quadratic extrapolation methods and the two‐voltage technique for the PTW Advanced Markus chamber are not statistically significant, and the trends of dose rate dependence are similar. The IBA PPC05 shows a flat response in terms of ion recombination effects based on the *k_s_* values calculated using the two‐voltage technique. Differences in *k_s_* values for the PPC05 between the two‐voltage technique and other model‐based extrapolation methods are not statistically significant at FLASH dose rates. Some of the *k_s_* values for the PPC05 that were extrapolated from the three‐voltage linear method and the semiempirical model were reported less than unity possibly due to the charge multiplication effect, which was negligible compared to the volume recombination effect in FLASH dose rates.

**Conclusions:**

The absolute dose measurements of both PTW Advanced Markus and IBA PPC05 chambers are in a good agreement with the National Physical Laboratory graphite calorimeter reference dose considering overall uncertainties. Both ion chambers also demonstrate good reproducibility as well as stability as reference dosimeters in UHDR PBS proton radiotherapy. The dose rate dependency of the ion recombination effects of both ion chambers in cyclotron generated PBS proton beams is acceptable and therefore, both chambers are suitable to use in clinical practice for the range of dose rates between 5 and 60 Gy/s.

## 1 INTRODUCTION

Recent preclinical studies showed that ultrahigh dose rate (UHDR) radiotherapy, or FLASH radiotherapy, may reduce normal tissue toxicity while maintaining tumor control.^1^, ^2^ Cincinnati Children's Hospital Medical Center and University of Cincinnati Medical Center Proton Therapy Center in collaboration with Varian Medical Systems, Palo Alto, CA, USA have enabled the dedicated research pencil beam scanning (PBS) gantry room for clinical FLASH proton radiotherapy. The Feasibility Study of FLASH Radiotherapy for the Treatment of Symptomatic Bone Metastases (FAST‐01),^3^ the first in‐human proton therapy FLASH clinical trial, began accruing patients in late 2020 and completed treating a cohort of 10 patients in October 2021.

In order to conduct a proton FLASH clinical trial, the absolute dose must be accurately and precisely measured. It is conventionally known that the charges collected from ion chambers exhibit dose rate dependence for a given dose. For parallel plate ion chambers, the charge collected, and therefore the dose measured, has primary dependence on two things—bias voltage (electric field inside air cavity) and incident dose rate. A reliable dose determination is a foundational factor on which all translational science and clinical trials rest. When using a dose‐to‐water formulism, based on an ion chamber Cobalt‐60 cross‐calibration, that dose determination relies on the reliable performance of the ion chamber.

The main purpose of this work is to compare the absorbed dose to water in UHDR (∼65 Gy/s) PBS proton beams measured with ion chambers and a graphite calorimeter from the National Physical Laboratory (NPL) in the United Kingdom as a reference. The charge collection efficiency in ion chambers should be assessed by dose‐rate‐independent systems. As calorimeters are dose rate independent and have a linear response with a given number of particles, it is a good reference dosimetry tool to determine the absolute dose in UHDR irradiation. The calorimeter‐based reference dose is also obtained from a direct measurement of absorbed dose, which is sufficiently accurate such that it is not calibrated by other standards. The dose determination from ion chambers was performed following IAEA TRS‐398 Code of Practice for Proton Beams Dosimetry protocol.^4^

Several studies exist devoted to assessing the collection efficiency of ion chambers either in UHDR proton beams^5^, ^6^, ^7^, ^8^, ^9^ or in conventional dose rate PBS.^10^, ^11^ However, to our knowledge, this is the first study to comprehensively and systematically evaluate the performance of commercially available, clinically ubiquitous plane‐parallel plate ion chambers in a PBS proton FLASH radiotherapy environment in support of FLASH clinical trials, and preclinical and translational science. Furthermore, this study seeks to evaluate various parameters impacting the collection efficiency and thereby the reference dose measurements, using practical and clinically applicable devices and methods, such as commonly available ion chambers and the widely used two‐voltage method.

## 2 METHODS AND MATERIALS

### 2.1 Absolute dose measurements and comparison

A total of eight rectangular 250‐MeV single‐layer transmission uniform fields (5 × 6, 5 × 8, 5 × 10, 5 × 12 cm^2^) and their transposed fields (6 × 5, 8 × 5, 10 × 5, 12 × 5 cm^2^) were developed for this study using a plateau region of the depth dose curves, similar to the treatment fields in FAST‐01. These predefined treatment plans were scaled to a physical dose of 7.64 Gy at isocenter in 5‐cm water‐equivalent depth (WED). Given that each field was a transmission field, delivering dose primarily on the lower LET entrance region of the Bragg peak, the RBE for these fields is set to 1.0, and so biological and physical doses are equivalent. Each spot of width 3.2‐mm sigma in air is separated by 5‐mm equal spacing. PBS dose rates were modeled at the isocenter plane in 5‐cm WED with a measured in‐water spot sigma of 3.65 mm, measured dose, and average spot irradiation times reported in the scanning nozzle system logfiles.^12^ The dose rate is ideally constant for these predefined treatment fields. Each field was designed to ensure that dose rate remains above 60 Gy/s.

The absolute dose measurements at 5‐cm WED for each field were performed to compare dose determination^4^ by two ion chambers and a graphite calorimeter. PTW (Physikalisch‐Technische Werkstaetten, Freiburg, Germany) Advanced Markus (1728) and IBA (Ion Beam Applications SA, Louvain‐la‐Neuve, Belgium) PPC05 (949) plane‐parallel plate chambers are our primary dosimeters with National Institute of Standards and Technology traceable calibration coefficients (*N_D_*_,_ *_w_ *= 1.521 Gy/nC and 0.608 Gy/nC, respectively) that are obtained from ^60^Co beam. Dose determination from both chambers with PTW T10010 UNIDOS E electrometer, in which the values of *k_s_* were determined from the two‐voltage technique following the IAEA TRS‐398 protocol,^4^ was compared with the absolute dose measured with a graphite calorimeter from the NPL in the United Kingdom as a dosimetric reference.

**FIGURE 1:**
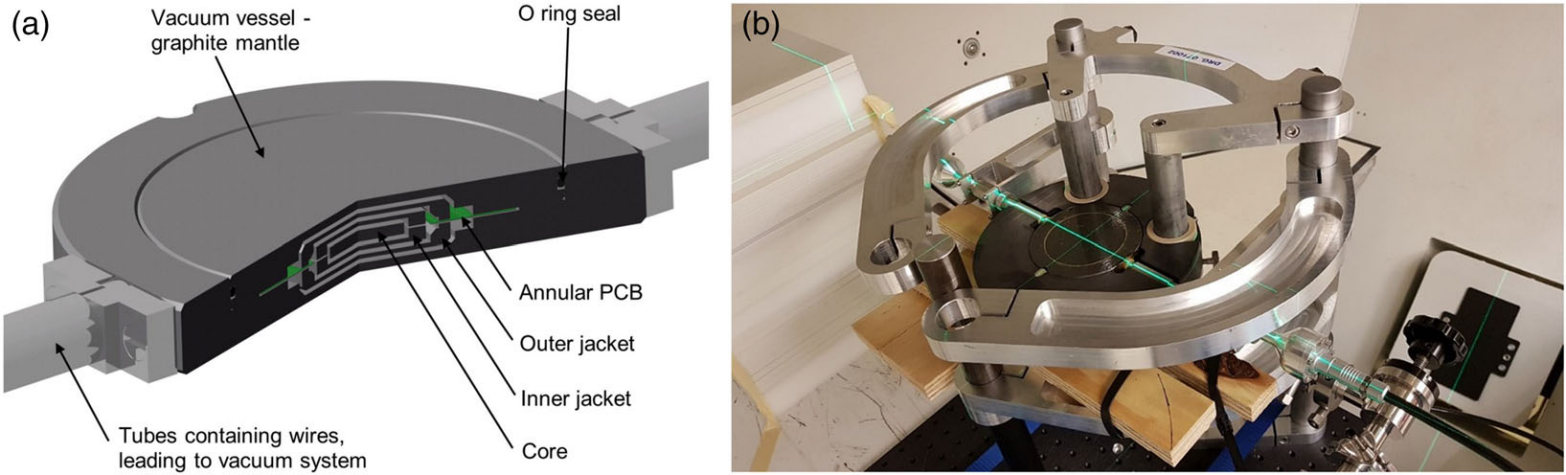
Schematic cross‐sectional image of the National Physical Laboratory (NPL) proton calorimeter from Ref. [17] (a) and NPL proton calorimeter aligned at isocenter at gantry zero at the Cincinnati Children's Hospital Medical Center‐University of Cincinnati Medical Center Proton Therapy Center (b)

The calorimeter consists of a series of graphite discs arranged in a nested construction and maintained under a high‐quality vacuum (Figure 1). Thermistors are equidistantly spaced and embedded around the circumference of each graphite component with each component connected to its own DC Wheatstone bridge, each bridge being monitored by a Keithley 2182A Nanovoltmeter. The calorimeter is operated in quasi‐adiabatic irradiation mode, and its thermistors detect small changes in the temperature of the graphite created by the energy absorbed from the radiation beam. From prior knowledge of the calibration coefficients of the thermistors and associated measuring system, and the specific heat capacity of the graphite, the dose absorbed by the graphite can be derived. The dose conversion between dose‐to‐graphite and dose‐to‐water was calculated using FLUKA v20221.2 Monte Carlo code.^13^ The two quantities are related by the fluence correction factor, $k_{fl}$, and the water‐to‐graphite stopping power ratio, $s_{w,g}$.^14^, ^15^ The simulated beam parameters (energy, energy spread, and divergency) were tuned against experimental data and graphite and water were defined according to recommendations of ICRU Report 90.^16^ The graphite mantle has a diameter of 100 mm, and the center of the graphite core of the calorimeter was positioned at the isocenter, and 5.9 g/cm^2^‐thick graphite plates with a diameter of 200 mm were placed in front of the calorimeter to position the graphite core at a WED of 5 g/cm^2^. A minimum of 20 irradiations were carried out for each radiation field allowing the mean and standard deviation of the mean (SDOM) dose from the calorimeter to be calculated.

### 2.2 Ion recombination measurements

For ion recombination measurements, the 5×12‐cm^2^ field was chosen from the predefined fields and delivered to the chamber at the same reference depth of 5 g/cm^2^. Bias voltages were varied from 50 to 400 V. For each setting, ion collection in the chamber was measured as a function of available dose rates over the range between 5 and 60 Gy/s, which are controlled by the nozzle currents as shown in Table 1. The dose rates were estimated by a voxel‐based dose rate calculation framework for PBS proton fields developed by Folkerts et al.^12^

**TABLE 1:** Mean values of voxel‐based dose rate calculations with standard deviation at various nozzle currents for the 5 × 12‐cm^2^ field

Nozzle current (nA)	20	40	70	80	105	135	160
Dose rate (Gy/s)	5.1 ± 0.3	10.6 ± 0.6	19.5 ± 1.0	30.8 ± 1.5	40.2 ± 1.7	52.1 ± 2.3	61.7 ± 2.9

The ion recombination effect in the ion chamber depends on the temporal structure of the proton beam. For the beam to be considered pulsed or pulsed‐scanned, the pulse duration must be short compared to ion transit time in the given volume of an ion chamber (∼10^−6^ s in Advanced Markus or PPC05), whereas the pulse‐to‐pulse interval or pulse repetition rate must be slow enough so that ionization events clear out among pulses. The ProBeam cyclotron produces 250‐MeV proton bunches with 0.2‐ns pulse duration at 72.8‐MHz RF repetition rate corresponding to 0.2‐ns micro‐pulses separated by 13.7‐ns intervals.^18^ For the case of a PBS system, the beam is delivered in so‐called spots where hundreds of pulses in a single spot occur during the ion transit time; each spot is delivered in relatively large time scales (10^−3^ s) and is then magnetically scanned across the plane perpendicular to the beam direction. Therefore, the ProBeam PBS beam may be approximated to be continuous regarding ion recombination effects.^10^, ^11^, ^19^

The values of *k_s_* factors were determined by the extrapolation technique based on Boag's theory.^20^, ^21^ In general, the collection efficiency is the ratio of collected charge $Q_{c}$ to produced or saturated charge $Q_{p}$ and the reciprocal of the collection efficiency is the $k_{s}$ factor:

(1) $$\;k_{s}=\frac{Q_{p}}{Q_{c}}\;\;.$$

In a continuous beam, if the collection efficiency is greater than 0.7, the recombination factor $k_{s}\;$can be written as^20^

(2) $$\;k_{s}=\;1+k_{c}\frac{Q_{p}}{V^{2}},$$

where the volume recombination dominates. The $k_{c}$ is an ion chamber–specific coefficient characterized by ion chamber dimension as well as charge density and mobility. Combining Equations (1) and (2), the produced charge $Q_{p}$ can be obtained by extrapolating measured values of the inverse of collected charges $1/Q_{c}$ as a linear function of the inverse square of polarizing voltages $1/V^{2}$,

(3) $$\;\frac{1}{Q_{c}}=\frac{1}{Q_{p}}\;+\frac{k_{c}}{V^{2}}.$$

In addition to the volume recombination, the initial recombination may play a role near the saturation, which is governed by a linear relationship of $1/Q_{c}$ and 1/V according to Jaffé’s model^22^ that saturates more slowly than the general recombination characterized by $1/Q_{c}$ as a linear function of $1/V^{2}$. A theoretical model of combining the first‐order term around 1/V=0 of Boag's and Jaffé’s models for continuous beams was developed by Niatel^23^:

(4) $$\;k_{s}=1+a\frac{1}{V}+k_{c}\frac{Q_{p}}{V^{2}},$$

where *a* is the chamber specific parameter. The produced charge $Q_{p}$ can be also obtained by extrapolating measured values of the inverse of collected charges $1/Q_{c}$ as a quadratic function of the inverse of polarizing voltages 1/V,

(5) $$\;\frac{1}{Q_{c}}=\frac{1}{Q_{p}}\;+\frac{a/Q_{p}}{V}+\frac{k_{c}}{V^{2}}.$$

The extrapolated values from the linear model (Equation 3) and the theoretical model (Equation 5) are then compared with the values of *k_s_* determined from the two‐voltage method (quadratic expression):

(6) $$\;k_{s}=\frac{{\left(\frac{V_{1}}{V_{2}}\right)}^{2}-1}{{\left(\frac{V_{1}}{V_{2}}\right)}^{2}-\frac{Q\left(V_{1}\right)}{Q\left(V_{2}\right)}}\;,$$

where $k_{s}$ is the recombination correction factor at the bias voltage of *V*_1_, and $\;Q(V_{1})$ and $Q(V_{2})$ are the collected charges at the bias voltages of $V_{1}=300\;V$ and $V_{2}=100\;V$, respectively. According to the IAEA TRS‐398 recommendation,^4^ the voltage ratio used in the measurement was 3.

The uncertainties of extrapolated saturated charge $Q_{p}$ from all models presented in this study are relative combined standard uncertainties of 1 standard deviation statistical uncertainties estimates from the model fits and standard errors from charge measurements. The uncertainties of *k_s_* obtained from the two‐voltage technique (Equation 6) and from all extrapolated models by using Equation (1) are derived from the error propagation principle.

## 3 RESULTS

### 3.1 Absolute dose measurements and comparison

The dose rate of all the predefined fields used for absolute dose measurements, which was designed to ensure that dose rate remains above 60 Gy/s, was estimated and confirmed by the voxel‐based dose rate calculation framework^12^ as ∼60 Gy/s. The provisional values of absolute doses to water measured by the NPL proton graphite calorimeter for each predefined field are shown in Table 2. The final calorimeter results with a more detailed uncertainty budget will be reported in another paper.^24^ The mean measured dose to water across all the predefined fields is 7.702 ± 0.037 Gy. The overall uncertainty includes type A and type B uncertainties. The repeatability of measuring the same quantity in the same conditions is considered type A, and all other uncertainties are grouped as type B as defined by the Guide to the Expression of Uncertainty in Measurement.^25^ Type A uncertainty is the standard error or SDOM and was of the order of 0.04% for each field. Type B uncertainties are comprised of several components, the largest of which are uncertainties related to the dose conversion between dose‐to‐graphite (quantity measured from the graphite calorimeter) and dose‐to‐water (quantity of interest) as well as uncertainties related to the determination of the specific heat capacity of graphite. A total uncertainty of 1.5% was estimated to 68% confidence level using a coverage factor of *k* = 1.

**TABLE 2:** Provisional values of absorbed dose to water measured by the National Physical Laboratory (NPL) proton graphite calorimeter

	NPL proton graphite calorimeter—provisional dose to water					
Field size (cm × cm)	5 × 6	5 × 8	5 × 10	5 × 12	6 × 5	12 × 5
Mean dose (Gy)	7.654	7.690	7.726	7.736	7.666	7.741
Overall expanded uncertainty, *k* = 1 (%)	1.50	1.50	1.50	1.50	1.50	1.50

The absorbed dose to water measured by clinically used plane‐parallel plate ion chambers, the PTW Advanced Markus and the IBA PPC05, following TRS‐398 protocol^4^ and the ratios of the absorbed dose determined with ion chambers to the absorbed dose measured with the NPL proton calorimeter are listed in Table 3. Measurement reproducibility with the standard error less than 0.7% was achieved for both chambers. The average ratio over the predefined fields of the PTW Advanced Markus chamber dose to the calorimeter reference dose is 1.002 ± 0.007, whereas the IBA PPC05 chamber shows a ∼3% higher reading of 1.033 ± 0.007.

**TABLE 3:** Absorbed dose to water measured by clinically used plane‐parallel plate ion chambers, Advanced Markus and PPC05, and the ratios of the absorbed dose determined with ion chambers to the absorbed dose measured with the National Physical Laboratory (NPL) proton calorimeter

Chamber	Field size (cm × cm)	Dose to water (Gy)	SDOM (Gy)	Ratio (chamber/calorimeter)	Average ratio
Advanced Markus	5 × 6	7.694	0.053	1.005 ± 0.007	1.002 ± 0.007
	5 × 8	7.710	0.054	1.003 ± 0.007	
	5 × 10	7.769	0.054	1.006 ± 0.007	
	5 × 12	7.701	0.054	0.995 ± 0.007	
	6 × 5	7.685	0.053	1.002 ± 0.007	
	12 × 5	7.746	0.054	1.001 ± 0.007	
PPC05	5 × 6	7.923	0.055	1.035 ± 0.007	1.033 ± 0.007
	5 × 8	7.954	0.056	1.034 ± 0.007	
	5 × 10	7.968	0.056	1.031 ± 0.007	
	5 × 12	7.971	0.056	1.030 ± 0.007	
	6 × 5	7.934	0.055	1.035 ± 0.007	
	12 × 5	7.991	0.056	1.032 ± 0.007	

Abbreviation: SDOM, standard deviation of the mean.

### 3.2 Ion recombination factor determination and comparison

**FIGURE 2:**
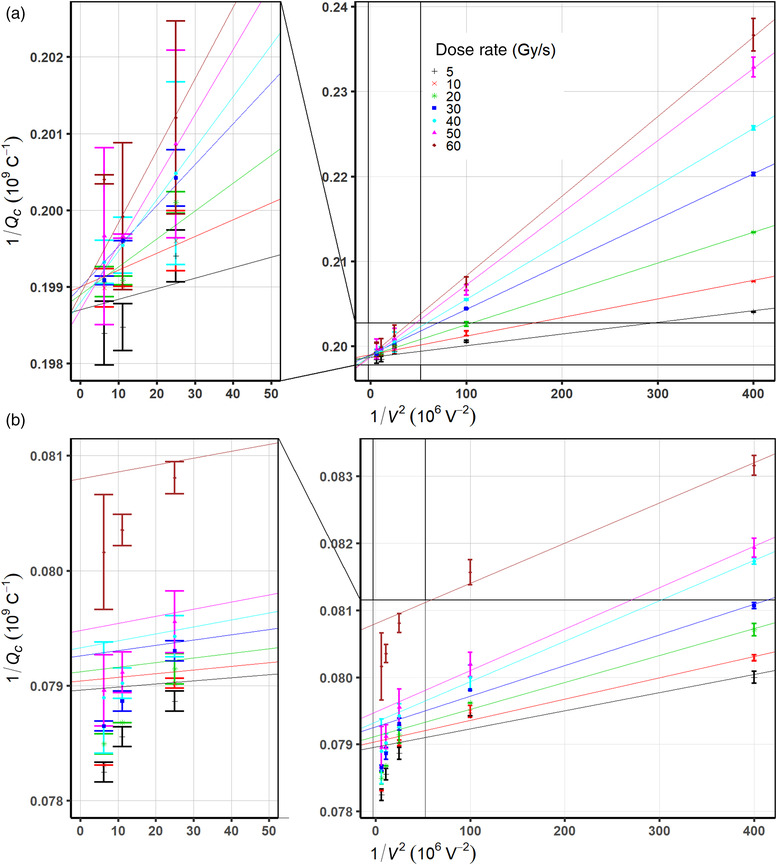
Measured data of the inverse of amount of collected charges 1/*Q* by the (a) Physikalisch‐Technische Werkstaetten (PTW) Advanced Markus and (b) the Ion Beam Application (IBA) PPC05 ion chambers, as a function of the inverse of the squared bias voltage 1/*V* ^2^ at various dose rates

Figure 2a,b illustrates the inverse of collected charge 1/*Q* versus the inverse of the squared bias voltages for the PTW Advanced Markus and the IBA PPC05 ion chambers, respectively, at various dose rates. As dose rate increased, the ion recombination effect increases for both ion chambers because the effect of volume recombination is larger. Ion recombination effects at higher dose rates are more pronounced at lower bias voltages. As seen in Figure 2a, for the PTW Advanced Markus chamber, small‐scale deviations from the linear fit exist; however, all lines converge onto the value of saturation charge $Q_{p}=\;5.030\pm 0.016\times 10^{-9}\;C$, which is the inverse of the extrapolated intercepts of each line from a linear regression model.

For the IBA PPC05, a nonlinearity of measured data points is observed in the high‐voltage region as shown in Figure 2b. Due to a systematic nonlinearity rather than random fluctuation in the saturation region, which leads to poor linear regression fit using the entire range of bias voltages, the linear extrapolation was performed in the linear region of low voltages (50, 100, 200 V) following a three‐voltage linear method suggested by Rossomme et al.^26^ The extrapolated saturation charge converges onto the value of $Q_{p}=\;12.591\pm 0.040\times 10^{-9}\;\;C$.

**FIGURE 3:**
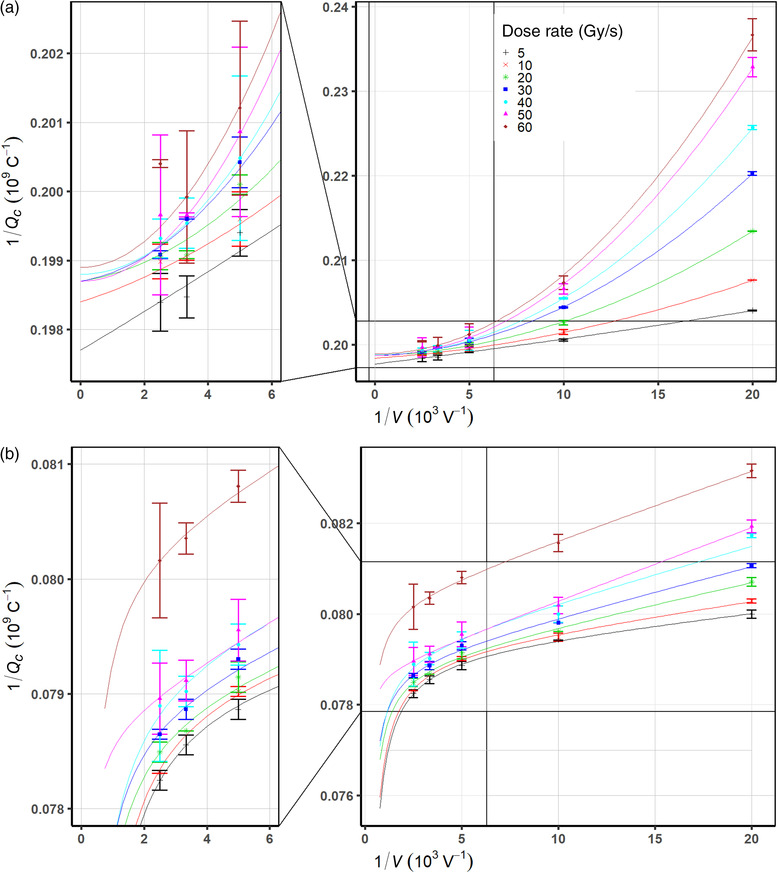
The inverse of collected charges versus the inverse of bias voltages at various dose rates fitted by the Niatel model for the Physikalisch‐Technische Werkstaetten (PTW) Advanced Markus (a) and the modified Niatel with charge multiplication correction for the Ion Beam Application (IBA) PPC05 (b) chambers

As the amount of the volume recombination effects is reduced with the decrease of dose rates, the initial recombination may become relevant. In order to evaluate the combined effects of initial and volume recombination for both chambers, a second‐order polynomial fit was performed to predict the saturated charge from a quadratic extrapolation using Equation (5). Figure 3a,b shows the inverse of collected charge 1/*Q* versus the inverse of the bias voltages for the PTW Advanced Markus and the PPC05 chambers. As shown in Figure 3a, the extrapolated values of the saturation charges for the PTW Advanced Markus chamber all converge onto $Q_{p}=\;5.033\pm 0.019\times 10^{-9}\;C$. The Niatel's quadratic model (Equation 5) based extrapolated value of the saturated charge accounting for both the initial and the volume recombination is 0.06% higher than the linear model (Equation 3) prediction for the Advanced Markus chamber, which neglects the initial recombination.

For the PPC05 chamber, no proper polynomial fits to all data points were found by using Equation (5). In other words, plotting 1/*Q* as a quadratic function of 1/V taking into account both initial and volume recombination effects did not explain the systematic excess charges across the range of investigated dose rates that appeared near the saturation voltage. A similar phenomenon was observed for 0.6‐cm^3^ Farmer chambers at voltages of 300–400 V in continuous ^60^Co beams, pulse linac beams,^27^, ^28^ and recently, plane‐parallel plate chambers under proton beam.^29^ This effect was assumed to be due to charge multiplication, which may be more significant for small volume chambers^30^ and was modeled by introducing an exponential function of bias voltages. In order to account for the charge multiplication and ion recombination together, the Niatel model (Equation 4) can be modified as

(7) $$k_{s}\cdot \;k_{s}^{CM}=\left(1+a\frac{1}{V}+k_{c}\frac{Q_{p}}{V^{2}}\right)\;e^{-\gamma V},$$

where $k_{s}^{CM}$ is the charge multiplication correction factor and γ is the charge multiplication parameter. Equation (5), thus, becomes

(8) $$\;\frac{1}{Q_{c}}=\left(\frac{1}{Q_{p}}+\frac{a/Q_{p}}{V}+\frac{k_{c}}{V^{2}}\right)\;e^{-\gamma V}.$$

Figure 3b shows a curve fit of measured data to Equation (8) accounting for charge multiplication in addition to the charge loss from initial and volume recombination. The curves agree with measured data very well in the whole range of polarizing voltages used to support the validity of combining the charge loss effects of initial and volume recombination with the charge‐gain effects of the charge multiplication. The saturation charge determined from the curve fit is $Q_{p}=\;12.627\pm 0.098\times 10^{-9}\;C$, which is 0.3% higher than the extrapolated saturation charge using the three‐voltage linear method. All extrapolated values of saturation charges for each chamber were listed in Table 4.

**TABLE 4:** The difference in the values of extrapolated charges from linear fit and curve fit along with standard errors for the Advanced Markus and the PPC05 chambers

	$Q_{p}$ from linear fit (nC)	$Q_{p}$ from curve fit (nC)	Difference (curve–linear, %)
Advanced Markus chamber (1728)	5.030 ± 0.016	5.033 ± 0.019	0.06±0.49
PPC05 (949)	12.591 ± 0.040	12.627 ± 0.098	0.29±0.84

**FIGURE 4:**
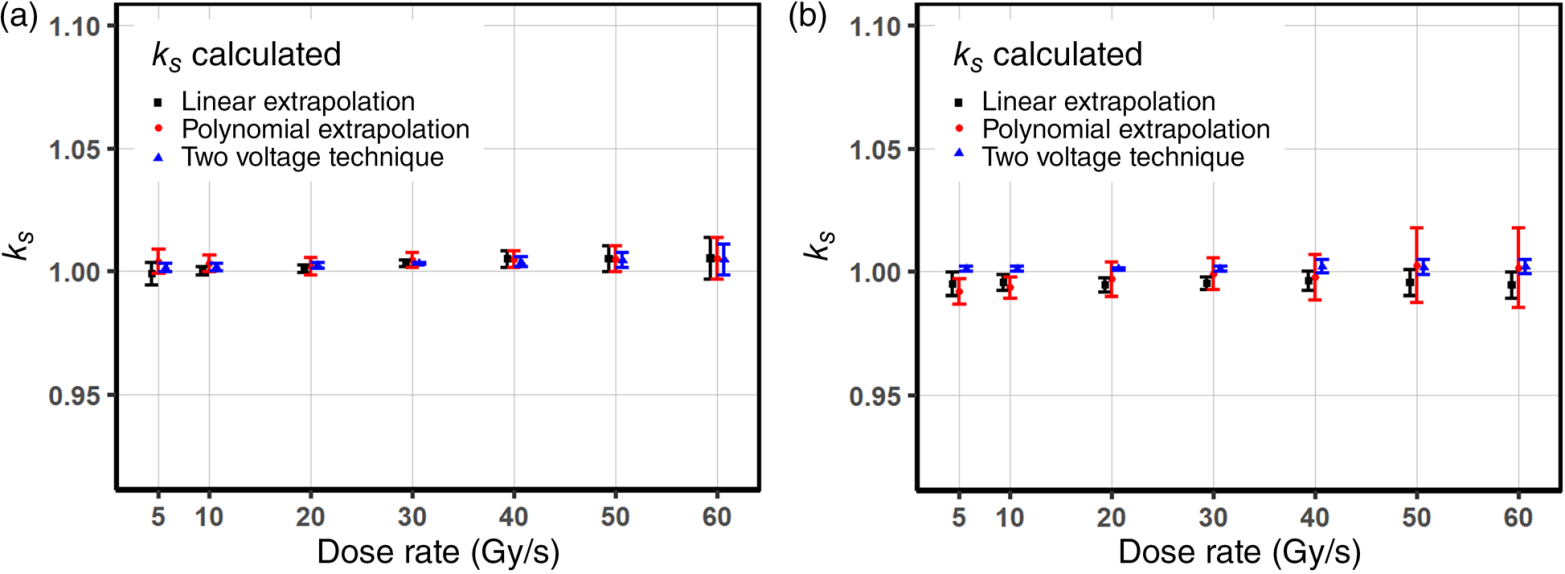
The extrapolated values of *k_s_* operated at 300 V and *k_s_* factors obtained using the two‐voltage technique at the voltage ratio of 3 for the Physikalisch‐Technische Werkstaetten (PTW) Advanced Markus (a) and the Ion Beam Application (IBA) PPC05 (b) chambers as a function of dose rates. Note that some of *k_s_* values from the linear and the semiempirical models are below unity after corrected for a possible charge multiplication effect

Figure 4a,b illustrates the extrapolated *k_s_* values for the Advanced Markus and the PPC05 chambers at bias voltage of 300 V as a function of dose rates. The *k_s_* values calculated using the two‐voltage technique (Equation 6) for the voltage ratio of 3 ($V_{1}=\;300\;V$ and $V_{2}=\;100\;\;V$) are added for comparison. For the Advanced Markus chamber as shown in Figure 4a, the relative difference in between the values from all three different methods agree within ∼0.5%. The *k_s_* values determined from the linear extrapolation and the polynomial methods are well matched in high‐dose rate region, and the quadratic model predicts higher *k_s_* values than the linear model, whereas the difference was pronounced in low dose rate region. The largest discrepancy of *k_s_* values (∼0.5%) was observed between linear and polynomial extrapolation methods; however, the difference is probably not statistically significant. The *k_s_* values from the two‐voltage technique based on the continuous beam formula differ by less than 0.3% compared to both extrapolated values throughout the entire range of investigated dose rates.

The IBA PPC05 in Figure 4b shows a relatively flat response in terms of ion recombination effects based on the *k_s_* values calculated using the two‐voltage method and the linear extrapolation. In other words, the changes of charge loss rates represented by the changes of slopes for each dose rate in Figure 2b are more gradual than the Advance Markus chamber as shown in Figure 2a. The three‐voltage linear extrapolation *k_s_* values are overall lower than the *k_s_* values from the two‐voltage technique up to ∼0.6%, where the extrapolated saturation charges are underestimated from the three‐voltage linear method as shown in Figure 3b. The extrapolated *k_s_* values from both models (Equations 2 and 7) appear to be less than unity, possibly due to the charge‐excess from the charge multiplication except for the high dose rate region over 50 Gy/s. In this region, the ion recombination correction factors from the semiempirical model (Equation 7) increase with dose rates, where the charge loss from the volume recombination dominates canceling out the charge gain effect from the charge multiplication in the region of high dose rates.

## 4 DISCUSSION

The results obtained in this study show that the ratio of doses determined with the PTW Advanced Markus and the IBA PPC05 parallel plate ion chambers to absorbed dose measured by the NPL proton graphite calorimeter are 1.002 ± 0.007 and 1.033 ± 0.007, respectively. The PTW Advanced Markus chamber dose measurement agrees with the calorimeter reference dose within 0.2%, whereas the IBA PPC05 chamber shows 3% higher readings. The discrepancy may be largely due to the uncertainty of the beam quality conversion factor *k_Q_*, which was estimated about 2.1% for plane‐parallel plate chambers according to IAEA TRS‐398 protocol.^4^ Considering an overall standard uncertainty of 2.3% (*k* = 1) for plane–parallel plate chambers for currently accepted IAEA TRS‐398 reference absorbed dose of proton beam measurement protocol and the NPL proton graphite calorimeter's overall uncertainty of 1.5% (*k* = 1), the 3% difference may not be statistically significant. The measured dose to water using the PPC05, thus, is still clinically acceptable, where determined doses to water by both ion chambers show a measurement reproducibility represented by standard error within 0.7%.

Under the irradiation of the ProBeam PBS proton beam considered a quasi‐continuous beam regarding ion recombination effects, both chambers show no evidence of loss of charge collection over the range of 5–60 Gy/s at bias voltages greater than 300 V. Although whether cyclotron generated PBS proton beams are considered pulsed or continuous is debatable,^31^ a rationale of our assumption that the ProBeam cyclotron generated PBS beam be continuous based on its pulse structure with the negligible initial recombination effect is confirmed by the linearity of the inverse values of charge readings as a function of the inverse of squared bias voltages as shown in Figure 2a for the PTW Advanced Markus chamber. Note that a recent study, including a Roos type plane‐parallel plate chamber under PBS proton beams, also showed that the initial recombination is comparable when dose rates are less than 2 Gy/s where the initial recombination effects are still only up to 0.04%.^11^ The inverse of collected charge as a function of the inverse of bias voltages at various dose rates is illustrated in Figure 3a. As voltage decreases the charge collection efficiency rapidly drops, which is more pronounced at higher dose rates. The data in Figure 3a were fitted with the Niatel's model (Equation 5) accounting for the initial recombination, which demonstrated the extrapolated saturation charge discrepancy between the linear (Equation 2), and the quadratic (Equation 4) models is ∼0.06% as shown in Table 4. The relative difference between the *k_s_* values determined using the two‐voltage technique and the *k_s_* values extrapolated using linear model (Equation 2) is negligible in high dose rates region over 40 Gy/s and becomes noticeable but less than 0.2% as shown in Figure 4a at lower dose rates. The *k_s_* values from the two‐voltage technique as well as the linear fit were also underestimated compared to the quadratic fit by up to 0.2% over the entire region of dose rates. These results are mainly because the initial recombination term is not properly dealt with in both the linear fit (Equation 2) and the two‐voltage technique formula for quasi‐continuous proton beams.^32^

The IBA PPC05 chamber shows a nonlinear behavior near high bias voltage region as shown in Figure 2b: systematic excess of collected charges across the range of dose rates. As the response of the PPC05 is more linear at low voltages, instead of using entire range of data points, the three‐voltage linear method provides a practical method to estimate the saturation charge so the *k_s_* value by allowing using three voltages in a linear region where charge multiplication is absent.^26^ The *k_s_* values from the three‐voltage linear method are constantly lower compared to the two‐voltage technique by up to 0.6%, even below unity as shown in Figure 4b. The linear relationship of $1/Q_{c}$ versus $1/V^{2}$ breaks down approaching near the saturation region leading to the underestimated prediction of the saturation charge $Q_{p}$ when the measured linear position of the $1/Q_{c}$ is extrapolated to $1/\;V^{2}=\;0$. In addition, an inadequate choice of the three voltages, which are in a nonlinear region (too high for charge multiplication or too low for higher order terms in both recombination), may produce inaccurate prediction of recombination correction factors. The *k_s_* values determined from the two‐voltage technique are consistently higher because a linear interpolation between two voltages (100 and 300 V), where the measured $1/Q_{c}$ at 300 V is lower (more measured charges at 300 V) than the three‐voltage linear fit line, extrapolating $1/Q_{c}$ to lower $1/Q_{p}$ of so the larger extrapolated saturation charge $Q_{p}$. This can be explained by a hypothesis of excess of collected charges near and at saturation region, which may be contributed from the non‐dosimetric process of charge multiplication.^27^, ^28^, ^29^, ^30^

No proper quadratic fits of the inverse of bias voltages to the inverse of collected charge data points, based on the Niatel model (Equation 5) accounting for the initial recombination, were found. This phenomenon was observed and confirmed with the charge multiplication model (Equation 7) by several early studies for photon beams.^27^, ^28^ This semiempirical model shows excellent agreement with the measured data near the saturation region supporting the hypothesis that the non‐dosimetric charge excess can be explained by the contribution from the charge multiplication process to the collected charge. Equation (7) implicitly contains the true produced charge $Q_{p}$, which can be extracted from the measured charges by separating the non‐dosimetric contribution of the charge multiplication from the dosimetric components. This charge multiplication effect becomes non‐negligible in small volume chambers where the electric field inside the air cavity increases.^30^ For example, the PPC05 produces the electric field strength of 500 V/mm at 300‐V bias voltage, which is almost as twice as in the Advanced Markus chamber. Recently, Rossomme et al. investigated the charge multiplication effect on the PPC05 chamber under quasi‐continuous PBS proton beam showing 1.5% overestimation of the two‐voltage method compared to the semiempirical model (Equation 7). Our study shows comparable results that *k_s_* values calculated from the two‐voltage method were overestimated by 1% in comparison with the *k_s_* values estimated from Equation (7). The discrepancy becomes smaller with dose rate approaching down to ∼0.03% at 60 Gy/s, where the volume recombination dominates the charge multiplication effect as well as the initial recombination effect.

The ion recombination factor *k_s_* (Equation 2) derived from the Boag's model is proportional to chamber dimension: $k_{s}∼d^{4}/v$, where *d* is the electrode spacing and *v* is the chamber volume and is dominated by the electrode spacing. As the plate separation of the IBA PPC05 chamber is almost half the PTW Advanced Markus chamber, the ions take less time to travel to electrodes and this reduces the probability of recombination. The IBA PPC05 chamber shows somewhat flat response as a function of dose rate where ion recombination correction factors have a small variation of *k_s_* ∼ 1.002 over the range of dose rates compared to the PTW Advanced Markus chamber *k_s_* values ranging from 1.001 to 1.006, which was confirmed by the two‐voltage technique as shown in Figure 4a,b. Given that the two‐voltage technique is based on the linear relationship between $1/Q_{c}$ versus $1/V^{2}\;$ for continuous beams, which is currently recommended by dosimetry protocols of AAPM TG‐51 and IAEA TRS‐398, its reliability for an accurate determination of $1/Q_{p}$, and subsequently the dose, may still be valid for UHDR PBS proton beams. However, when using submillimeter‐scale small ion chambers at critical, high voltages under low conventional dose rate proton beams, those ion chambers may cause a non‐negligible over‐response due to the charge multiplication process, which needs to be thoroughly investigated and corrected for.

This study was primarily designed to support the FAST‐01 FLASH clinical trial and thus, several experimental parameters such as single energy, predefined transmission fields, doses, and dose rates were limited by the FAST‐01 protocol. More extensive investigation on ion recombination with a considerable amount of data measurements for accurately modeling non‐dosimetric components such as the charge multiplication will be a future study. In addition, recombination study can be substantially different in the spread‐out Bragg peak FLASH as the dose rates as well as LETs vary along the depth and are higher in the Bragg peak and distal edge, which requires a substantial number of measurement points such as measurements along a depth dose distribution.

For FLASH radiotherapy, there still exists a heterogeneity in the definition of dose rate. For this study, the dose rate definition, called local or neighborhood dose rate, is based on a published framework^12^ directly applicable for PBS but generalizable to other modalities. In the assessment of ion chamber collection efficiency and recombination, it is adequate to use any dose rate definition, as long as the definition translates to the clinical application. However, when translating these results to the performance of ion chambers in other beam delivery systems or modalities, care should be taken. Ion recombination effects are impacted by the radiation modality or particle, pulse structure, dose rate, chamber geometry, and so forth. For example, a transmission PBS proton beam at 60 Gy/s, with that dose rate defined by Folkerts et al.^12^ dose rate framework, is not the same as a pulsed electron beam or even a scattered proton beam at 60 Gy/s.

## 5 CONCLUSIONS

This study carried out a dosimetric comparison between the NPL proton graphite calorimeter with the PTW Advanced Markus and the IBA PPC05 plane‐parallel plate chambers and their recombination effects in UHDR PBS proton beams as support of first FLASH human clinical trial (FAST‐01). The PTW Advanced Markus chamber dose measurements agree with the NPL graphite calorimeter reference dose within 0.2%, whereas the IBA PPC05 chamber shows 3% over‐response, which is clinically acceptable considering overall uncertainties in ionometric (2.3%) and calorimetric (1.5%) methodologies. Both ion chambers also demonstrate good reproducibility as well as stability as reference dosimeters in UHDR PBS proton radiotherapy.

The investigation of the ion recombination effect of both chambers at various dose rates was also undertaken. At reference bias voltage of 300 V, the ion correction factors calculated using the two‐voltage technique for a continuous beam match the values determined from the extrapolation methods within 0.3%, and the dose rate dependency of all *k_s_* values from three different methods is less than 0.5% over the range of 5–60 Gy/s for the PTW Advanced Markus chamber. The IBA PPC05 recombination correction factor for PBS proton beams, based on the two‐voltage technique for a continuous beam, is approximately 1.0% overestimated at a dose rate of 5 Gy/s compared to the charge multiplication–corrected *k_s_* values estimated using the semiempirical model, but no statistically significant difference in FLASH dose rates region. Therefore, both chambers are suitable to be used in cyclotron‐generated FLASH PBS systems.

## CONFLICT OF INTEREST

Richard A. Amos is on the Clinical Advisory Board of TAE Life Sciences.

## Data Availability

Some of the data that support the findings of this study are available from the corresponding author upon reasonable request. Otherwise, the authors elect to not share data.
